# Risk assessment of disease recurrence in early breast cancer: A serum metabolomic study focused on elderly patients

**DOI:** 10.1016/j.tranon.2022.101585

**Published:** 2022-11-17

**Authors:** Emanuela Risi, Camilla Lisanti, Alessia Vignoli, Chiara Biagioni, Agnese Paderi, Silvia Cappadona, Francesca Del Monte, Erica Moretti, Giuseppina Sanna, Luca Livraghi, Luca Malorni, Matteo Benelli, Fabio Puglisi, Claudio Luchinat, Leonardo Tenori, Laura Biganzoli

**Affiliations:** a Sandro Pitigliani Medical Oncology Department, Hospital of Prato, Prato, Italy; b Cro Aviano - National Cancer Institute - IRCCS, Medical Oncology and Cancer Prevention, Aviano, Italy; c Magnetic Resonance Center (CERM), University of Florence, Sesto Fiorentino, Italy; d Bioinformatics Unit, Hospital of Prato, Prato, Italy

**Keywords:** Metabolomics, Breast cancer, Prognosis, NMR spectroscopy, Elderly, eBC, early breast cancer, aBC, advanced breast cancer, BC, breast cancer, 1H NMR, proton nuclear magnetic resonance, NMR, nuclear magnetic resonance, RF, Random Forest, TN, triple negative, HR, hazard ratio, CI, confidence interval, GCP, good clinical practice, CGA, comprehensive geriatric assessment, AJCC, American Joint Committee on Cancer, PQN, probabilistic quotient normalization, FFDR, free from disease relapse, ROC, receiver operating characteristic, RFI, recurrence-free interval, OS, overall survival, AUC, area under the curve, T, tumor stage, N, lymph nodal stage

## Abstract

**Background:**

We previously showed that metabolomics predicts relapse in early breast cancer (eBC) patients, unselected by age. This study aims to identify a “metabolic signature” that differentiates eBC from advanced breast cancer (aBC) patients, and to investigate its potential prognostic role in an elderly population.

**Methods:**

Serum samples from elderly breast cancer (BC) patients enrolled in 3 onco-geriatric trials, were retrospectively analyzed via proton nuclear magnetic resonance (1H NMR) spectroscopy. Three nuclear magnetic resonance (NMR) spectra were acquired for each serum sample: NOESY1D, CPMG, Diffusion-edited. Random Forest (RF) models to predict BC relapse were built on NMR spectra, and resulting RF risk scores were evaluated by Kaplan–Meier curves.

**Results:**

Serum samples from 140 eBC patients and 27 aBC were retrieved. In the eBC cohort, median age was 76 years; 77% of patients had luminal, 10% HER2-positive and 13% triple negative (TN) BC. Forty-two percent of patients had tumors >2 cm, 43% had positive axillary nodes. Using NOESY1D spectra, the RF classifier discriminated free-from-recurrence eBC from aBC with sensitivity, specificity and accuracy of 81%, 67% and 70% respectively. We tested the NOESY1D spectra of each eBC patient on the RF models already calculated. We found that patients classified as "high risk" had higher risk of disease recurrence (hazard ratio (HR) 3.42, 95% confidence interval (CI) 1.58–7.37) than patients at low-risk.

**Conclusions:**

This analysis suggests that a “metabolic signature”, identified employing NMR fingerprinting, is able to predict the risk of disease recurrence in elderly patients with eBC independently from standard clinicopathological features.

## Highlights

• We reported the results of a retrospective study performed on elderly breast cancer patients enrolled in 3 onco-geriatric trials coordinated by the medical oncology division of the Hospital of Prato. This analysis suggests that a metabolomic signature, identified employing NMR spectral profiling, is able to predict the risk of disease recurrence in elderly patients with early breast cancer, independently from classical clinicopathological features. The metabolomic signature is a prognostic marker that takes into account not only the tumor but also the host. This is particularly relevant in the elderly, due to the heterogeneity of this population, and particularly in older patients with early breast cancer in whom other causes of death can compete with breast cancer in determining patients’ outcome.

## Introduction

Over 40% of patients with BC are aged ≥65 years at diagnosis, while approximately 20% are older than 75 years [1]. BC correlation with age could be linked to the continuous endocrine proliferative stimulus that the mammary epithelium undergoes over the years, as well as to the progressive damage of DNA and the accumulation of epigenetic mutations that alter the balance of expression between oncogenes and tumor suppressor genes [2]. More favorable breast cancer subtypes are prevalent in older women with around 80% of BC occurring in the elderly being hormone dependent and weakly aggressive [3]. Despite the apparent favorable biological characteristics, BC-specific mortality rates increase with age [4]. This is mostly related to undertreatment, as well as to the heterogeneity of the elderly population [5]. Management of older cancer patients is challenging due to limited level I evidence to guide treatment decisions. Older patients are generally underrepresented in clinical trials, therefore treatment recommendations are largely based on retrospective subgroup analysis and extrapolation of study results from younger individuals [6,7]. However, it is not always possible to translate evidence derived in the younger to the older population, mainly because of a different “habitus” that can condition treatments’ response, tolerability and compliance. Moreover, older patients enrolled in clinical trials, are not representative of the “real” population because of selection process. Selection bias is common also in elderly-focused clinical trials, in which unfit patients are scarcely represented.

Aging is associated with decreased physiological reserves and altered pharmacokinetics, influencing cancer treatment toxicity, response, and overall prognosis [8,9]. Therefore, it is important to evaluate the cost/benefit ratio of an anticancer treatment in each older cancer patient. Prognostic factors that consider not only the tumor but also the host, and a better definition of patient health status, could help in identifying the patients that will benefit most from the treatment and those who may need a personalized approach.

In the treatment of eBC, risk stratification based on prognostic features is critical to decisions about the appropriate adjuvant strategies. In the past years, molecular profiling of the primary tumor has led to an improvement of the traditional pathological risk stratification [10], [11], [12]. However, a significant proportion of patients classified as “high risk” by clinicopathological factors and/or by genomic analysis, do not relapse and may receive chemotherapy unnecessarily. In addition to primary cancer characterization, the detection of micrometastatic disease should contribute to a more precise definition of the risk of relapse.

Metabolomics is the omics science that deals with the characterization of the metabolome, that is a highly complex and organized biochemical network in which small molecules such as lipids, peptides, vitamins and other cofactors, are released in biological specimens (i.e. blood, urine, tissue) and interact between them and with other biological macromolecules [13]. Metabolomics offers a major opportunity in identifying individual susceptibility to drugs and environmental factors, as well as in characterizing a disease for diagnostic and prognostic purposes; metabolomics can also be useful for understanding the biochemical causes underlying different pathophysiological conditions [14]. Given that cancer cells can significantly alter metabolism, the pattern of metabolites produced can yield a “signature” that may indicate the cancer's presence or behavior [15]. In contrast to gene expression profiling, metabolomics identifies a signal that originates not only from the primary tumor tissue, but also potentially from the micrometastatic disease [16,17]. Furthermore, factors derived from the interaction between the cancer and the individual (i.e. tumor micro-environment, inflammatory and immune responses), may contribute to the metabolomic profile, offering combined information on residual tumor and host response. Previously published data from our group have shown that metabolomic spectra derived using NMR to analyze the metabolites in serum samples, can discriminate patients with eBC from those with aBC, and that patients with eBC who have metabolomic spectra more resembling the metastatic profile are more likely to relapse [18], [19], [20]. Due to the heterogeneity of the older population we cannot speculate that the findings observed in a younger population can be translated to the elderly.

The aim of the present study is to compare the NMR spectra of serum samples from elderly eBC patients with elderly aBC patients, to identify a “metabolic signature” that could differentiate these two groups. In addition, we would investigate the prognostic role of this metabolic model in terms of disease recurrence and survival.

## Methods

This retrospective study was a collaborative project between the Sandro Pitigliani Medical Oncology Department, Hospital of Prato (Prato, Italy) and the Magnetic Resonance centre of the University of Florence (Sesto Fiorentino, Italy). Trials included in these analyses received approval from the local institutional ethics committee and were conducted in accordance with good clinical practice (GCP) and the principles of the Declaration of Helsinki. Written informed consent was prospectively obtained from all patients participating in these trials.

### Patient selection

Serum samples of elderly BC patients enrolled in three onco-geriatric trials coordinated by the Medical Oncology Division of the Hospital of Prato were retrospectively analyzed.

The “MetaboGER” study [21] is a prospective trial that has evaluated the performance of different geriatric assessments in identifying frailty, in elderly patients with solid tumors. The “GIVE” trial [22] [NCT02785887], is a randomized multicentric trial that aims to determine the impact of Comprehensive Geriatric Assessment (CGA)-based interventions on chemotherapy delivery, in patients aged 70 years or older, who present at least one deficit identified at a CGA. “CAFFE” is a complementary study to GIVE and aims to evaluate chemotherapy compliance in “fit” older patients.

All three trials targeted patients with early stage or advanced solid malignancies (including breast cancer), who underwent a CGA at study entry. In GIVE and CAFFE, enrolled patients received chemotherapy as adjuvant treatment, or first/second line therapy for advanced disease.

For the present analysis, the inclusion criteria for the “eBC cohort” were: age ≥70 years, diagnosis of early-stage BC (stage I-III according to American Joint Committee on Cancer (AJCC) 2017 classification [23]), radical surgical treatment of primary BC, availability of a fasting peripheral blood sampling performed within a maximum of 3 months from the date of breast surgery and before starting any type of medical cancer treatment in the adjuvant setting. Likewise, the inclusion criteria for the “aBC cohort” were: age ≥70 years, diagnosis of advanced BC (stage IV according to AJCC 2017 classification [23]), availability of a fasting peripheral blood sampling performed before starting medical cancer treatment for advanced disease.

### Serum sample collection and storage

For each patient, an overnight fasting peripheral blood sample (10 mL) was collected after surgery, and prior to commencement of the systemic treatment. Blood was centrifuged at room temperature for ten minutes at 1500 g, then serum was collected, and 1 mL aliquots transferred into pre-labelled cryovials. Within one hour of collection, samples were frozen and then stored at −80 °C pending NMR analysis.

### NMR analysis

Serum samples were prepared according to the standard operating procedures developed in the CERM laboratory [14,24]. One-dimensional 1H NMR spectra were acquired using a Bruker 600 MHz spectrometer (Bruker BioSpin, Rheinstetten, Germany) operating at 600.13 MHz proton Larmor frequency. Before measurement, for temperature equilibration at 310 K, samples were kept for at least 5 min inside the NMR probe head.

For each serum sample, three one-dimensional 1H NMR spectra were acquired using three different pulse sequences that enable the selective detection of different molecular components: a standard nuclear Overhauser effect spectroscopy pulse sequence NOESY 1D presat was applied to detect both signals of low (metabolites) and high molecular weight molecules. A standard spin echo Carr-Purcell-Meiboom-Gill 1D sequence (CPMG) and a standard diffusion-edited pulse sequence were used to selectively detect signals of low molecular weight metabolites and high molecular weight macromolecules (e.g. lipoproteins, proteins), respectively.

An extended description of the sample preparation procedures, instrument configuration, and setting of the NMR parameters can be found in our previous publication [19].

### NMR spectra processing

Free induction decays were multiplied by an exponential function equivalent to a 1.0 Hz line-broadening factor before applying Fourier transform. Transformed spectra were automatically corrected for phase and baseline distortions and calibrated (anomeric glucose 1H doublet at δ 5.24 ppm) using TopSpin 3.6.2 (Bruker Biospin).

### Statistical analysis

All data analysis was performed using the open source “R” statistical environment (Version Microsoft R Open 4.0.2). Multivariate analysis was performed on bucketed spectra. Each 1D spectrum in the range between 0.2 and 10.0 ppm was segmented into 0.02 ppm chemical shift bins and the corresponding spectral areas were integrated using AssureNMR software (Bruker BioSpin). The spectral region containing residual water signal (5.10–4.42 ppm) was removed and the dimension of the system was reduced to 456 bins. Probabilistic Quotient Normalization (PQN) was applied on the data prior to pattern recognition [25]. Since spectra were acquired in a timeframe of 10 years (2008–2018) we needed to remove from the NMR data differences associated to technical variations. To this aim, we used a regression approach already described in previous publications [26,27].

A RF classifier [28] was built to separate eBC free from disease relapse (FFDR) patients, unselected for molecular subtypes, from aBC patients, as reported in previous studies [18,19,29]. RF is a classification algorithm that uses an ensemble of unpruned decision trees (forest), each of which is built on a bootstrap sample of the training data using a randomly selected subset of variables (bins in the present model) [30,31,32]. We split our cohort in two independent cohorts: a training set consisting of 111 eBC patients recurrence-free at follow up plus all aBC patients, and a validation set consisting of all relapsed eBC patients (29 subjects). The initial analysis was restricted to the training set. In our RF model each tree is used to predict whether a sample come from an eBC patient recurrence-free at follow up or from an aBC patient. For each eBC patient, a score was created that expresses the extent to which the serum metabolomic fingerprint appeared to be similar (percentage of trees in the ensemble that misclassify the sample as belonging to the cohort of aBC patients) to the profile of a confirmed metastatic sample, designated as the ‘RF risk score’. RF models were built using the R package Random Forest with default settings [33]. Separate models were built for NOESY1D, CPMG and Diffusion edited spectra. Receiver Operating Characteristic (ROC) curve analyses were used to evaluate the performance of the RF classifier in discriminating early and advanced BC. Using the same methodology, we built an RF classifier focused only on luminal eBC patients.

The second step was to test the hypothesis that metabolomic classification of some eBC patients as metastatic was due to metabolomic detection of signals from micrometastatic disease with likelihood for tumor relapse. For this aim, the NMR spectra of each relapsed eBC patient was tested on the RF models already calculated (we assumed that higher RF scores correlated with higher risk of developing cancer relapse). If a sample was classified as metastatic, the patient was considered at high risk of BC recurrence. BC recurrence was defined as tumor loco-regional or distant recurrence or development of a contralateral BC. The recurrence-free interval (RFI) was defined as the time interval between the date of informed consent and BC recurrence or BC-related death. BC recurrence alone, overall survival (OS), and BC-specific survival, were also evaluated. We considered a death related to BC if the patient had already developed a disease relapse.

The ability of the RF risk score to predict BC relapse was assessed using Kaplan–Meier curves (R packages “survival” and “survminer”) with additional calculation of the HR and p-value assessed by Log-Rank test.

The spectral region related to 28 different metabolites was quantified by using a R script developed in-house. Multivariable logistic regression models were calculated to estimate the association between metabolites and BC stage (early free from disease relapse vs. metastatic patients) and adjusted for the time of serum sample acquisition [26,27]. Logistic regression models were computed using the function “glm” (R package “stats”) and each model significance was assessed through a Wald test. The *p*-values were corrected for multiple testing using the false discovery rate procedure with Benjamini-Hochberg correction at α = 0.05.

## Results

Serum samples from 140 women with eBC and 27 with aBC, collected between November 2008 and August 2018, were retrieved. The median age at study entry was 79 (range 70–88) years in the aBC cohort, and 76 (range 72–80) years in the eBC cohort. In the aBC cohort, 13 patients (48%) had luminal BC, 8 (30%) HER2-positive BC, and 6 (22%) TN BC. The major clinicopathological characteristics of the eBC cohort are reported in Table 1. Eighty-one patients (57%) had pT1 and 58 (42%) had pT2 tumors; axillary-nodes involvement was present in 60 patients (43%). The majority of patients (77% *n* = 108) had luminal (hormone receptor positive, HER2-negative) BC; while 10% and 13% presented a HER2-positive and TN BC, respectively. Adjuvant systemic therapies were so distributed: 15 patients (11%) received only chemotherapy, 30 patients (21%) received chemotherapy and endocrine therapy, 80 patients (57%) received only endocrine therapy, 15 patients (11%) received no adjuvant treatment. At a median follow-up of 9.6 years (95% CI 9.1–9.8), 29 patients (21%) had disease relapse, 61 patients (44%) had died, of which 26 patients (43%) were tumor related (21 out of 26 patients had a distant recurrence before death). RFI probability at 5-years was 81% (95%CI 74%−88%), while the RFI probability at 10-years was 74% (95%CI 67%−83%). Median-OS was 9.5 years (95%CI 8.5–10 years).

**Table 1:** Baseline characteristics of the eBC cohort.

	Overall population *N* = 140 (%)
Study	
CAFFE	23 (16%)
GIVE	12 (9%)
METGER	105 (75%)
**Age at study entry**	
Median (Q1, Q3)	76 (72, 80)
Range	70 - 91
**Gender**	
F	136 (97%)
M	4 (3%)
**Pathologic T stage**	
pT1	81 (58%)
pT2	59 (42%)
**Pathologic N stage**	
pN0	79 (56%)
pN1	38 (27%)
pN2	10 (7%)
pN3	12 (9%)
pNx	1 (1%)
**Histological types**	
Ductal	122 (87%)
Lobular	11 (8%)
Other*	7 (5%)
**Histological grade**	
G1	24 (17%)
G2	76 (54%)
G3	40 (29%)
**Ki67**	
<20	45 (32%)
>=20	95 (68%)
**Vascular invasion**	
No	83 (59%)
Yes	57 (41%)
**Molecular subtypes (by IHC)**	
Luminal (ER+ and/or PR+ HER2-negative)^⁎⁎^	108 (77%)
HER2-positive	14 (10%)
Triple negative	18 (13%)
**Adjuvant therapy**	
CT and ET	30 (21%)
CT only	15 (11%)
ET only	80 (57%)
No treatment	15 (11%)

*F*= female, *M*=male, *T*= tumor stage, *N*= lymph nodal stage, *G*= tumor grade, IHC = immunohistochemistry, ER= estrogen receptor, PR= progesterone receptor, eBC= early breast cancer, CT= chemotherapy, ET= endocrine therapy

*Other=no special type invasive carcinomas, mixed ductal-lobuar carcinomas

^⁎⁎^ER+= ER ≥1%; PR+= PR ≥1%.

### Metabolomic discrimination between non-relapsed eBC and aBC patients

**Fig. 1:**
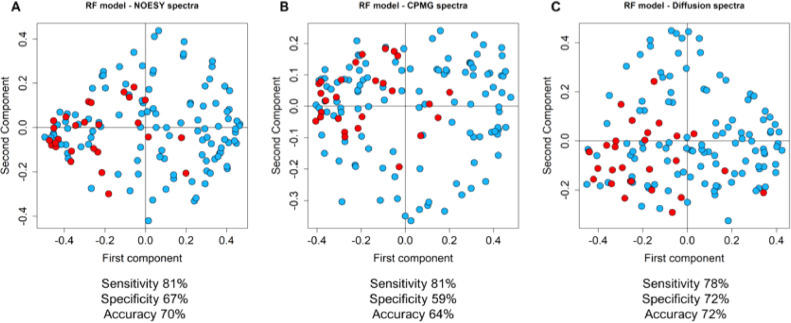
Proximity plots of the RF models discriminating advanced (red, *n* = 27) and early FFDR (light blue, *n* = 111) BC patients on the base of their serum metabolomics fingerprint using (A) NOESY1D; (B) CPMG; (C) Diffusion NMR spectra.

One-dimensional NOESY1D, CPMG and DIFFUSION-edited 1H—NMR spectra were acquired for all serum samples. Supervised analysis, using RF classifier, showed significant differential clustering (permutation test *p-*value = 0.01 for all models) between serum spectra of early patients FFDR unselected for molecular subtypes, and metastatic patients. Sensitivity, specificity, and accuracy were respectively 81%, 67% and 70% for the NOESY1D spectra (Fig. 1A), 81%, 59% and 64% for the CPMG spectra (Fig. 1B), and 78%, 72% and 72% for the DIFFUSION-edited spectra (Fig. 1C).

Using ROC analyses, an area under the curve (AUC) scores of 0.79, 0.78, and 0.81 were obtained for NOESY1D, CPMG and DIFFUSION-edited spectra, respectively (supplementary figure 1).

We conducted a subgroup analysis in patients with luminal tumors. In this subgroup, the RF model, built using the NOESY spectra, discriminated eBC and aBC with 69% sensitivity, 66% specificity, 66% accuracy and an AUC of 0.706. The statistical significance was not reached, mainly due to the small number of luminal patients (*n* = 13) included in the aBC cohort (data not shown).

Twenty-eight metabolites were quantified in all serum spectra. From the logistic regression analysis emerged that higher serum levels of 3-hydroxybutyrate, citrate, N,N-Dimethylglycine and phenylalanine and lower serum levels of leucine, isoleucine, valine, acetate and histidine were associated with the presence of aBC as compared with eBC FFDR (supplementary figure 2). No statistically significant correlation was found between the levels of individual metabolites and the risk of recurrence in the whole eBC cohort (supplementary figure 3). Interestingly, we found that all except two (phenylalanine and acetate) metabolites significantly associated with aBC, had the same trend in the relapsed eBC.

### Relapse prediction by RF risk score

We compared metabolomic RF risk scores and actual BC recurrence. Using NOESY1D, 19 out of 29 recurred patients were classified as “high risk”, meaning that 66% of patients with BC recurrence in the eBC cohort were predicted as “metastatic-like” based on the resemblance with the profiles of the metastatic samples. Worse ability to predict BC recurrence was seen for CPMG (15 out of 29 (51.7%) recurred patients correctly predicted), and for DIFFUSION-edited (12 out of 29 (41.3%) recurred patients correctly predicted). Therefore, we selected the NOESY1D NMR spectra for the subsequent analysis. Respectively 84 and 56 out of 140 eBC patients (60% and 40%) were classified as “low risk” or “high risk” by their metabolomic fingerprint. Major clinicopathological features of eBC patients by metabolomic risk score are reported in supplementary Table 1. Ten patients (12%) of the “low risk” group presented tumor recurrence, versus 19 (34%) of the “high risk” metabolomic group.

**Fig. 2:**
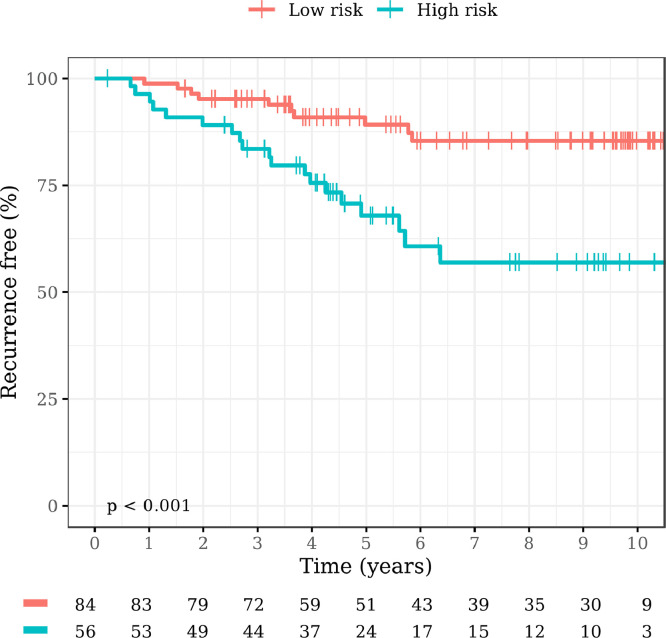
Kaplan-Meier plot of RFI by metabolomic risk score.

As shown in Fig. 2, patients classified as "high risk" (red – RF clas=aBC) had a higher risk of BC recurrence than patients classified as "low risk" (light blue – RF clas=FFDR eBC) (HR 3.42, 95% CI 1.58–7.37).

The same analysis was also repeated excluding patients with second metachronous ipsilateral or contralateral BC. Even in this case, patients classified by the model as "high risk" had a higher risk of BC recurrence than patients classified as "low risk" (HR 2.98, 95% CI 1.33–6.66, *p* = 0.0044) (data not shown).

The metabolomic risk score and conventional clinicopathological prognostic factors, were examined by univariate and multivariate regression analysis. The metabolomic risk score (*p* = 0.002), tumor (T) stage (*p* = 0.003), lymph nodal (N) stage (*p*<0.001), TN and luminal subtypes defined by IHC (*p* = 0.004), were significantly associated with the risk of recurrence at univariate analysis. The multivariate analysis showed that the prognostic effect of the metabolomic classifier was independent of pathological N stage, pathological T stage, and molecular subtypes (Table 2).

**Table 2:** RFI uni/multivariate.

	Univariate			Multivariate		
	HR	95% CI	p-value	HR	95% CI	p-value
**Metabolomic risk** High vs Low	3.39	1.57–7.33	0.002	2.96	1.35–6.48	0.01
**T stage** T2 vs T1	3.18	1.47–6.85	0.003	1.76	0.77–4.05	0.2
**N stage***N*+ vs N0	4.07	1.80–9.21	<0.001	3.18	1.32–7.66	0.01
**Tumor grade** G2–3 vs G1	2.44	0.74–8.10	0.14			
**Molecular subtypes** Her2 pos vs Lum	0.99	0.23–4.28	>0.9	0.6	0.14–2.67	0.5
**Molecular subtypes** TN vs Lum	3.69	1.54–8.89	0.004	3.54	1.41–8.91	0.01

HR= Hazard Ratio, CI= Confidence Interval, T stage= tumor stage, N stage= lymph-nodal stage, Lum= luminal, TN=triple negative, pos=positive, RFI= recurrence free interval.

**Fig. 3:**
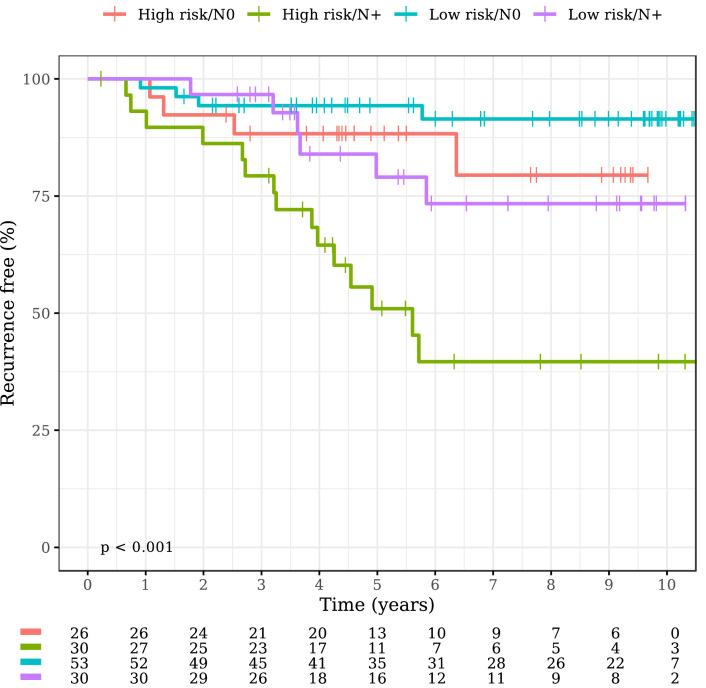
Kaplan-Meier plot of RFI by metabolomic score and nodal status.

RFI by metabolomic score and lymph node status is reported in Fig. 3. A significant difference was observed between high and low metabolomic risk in patients with node positive tumors (High risk/*N*+ vs. Low risk/*N*+ HR 3.05, 95%CI 1.18–7.88, *p* = 0.022). No difference was found in the node negative group.

**Fig. 4:**
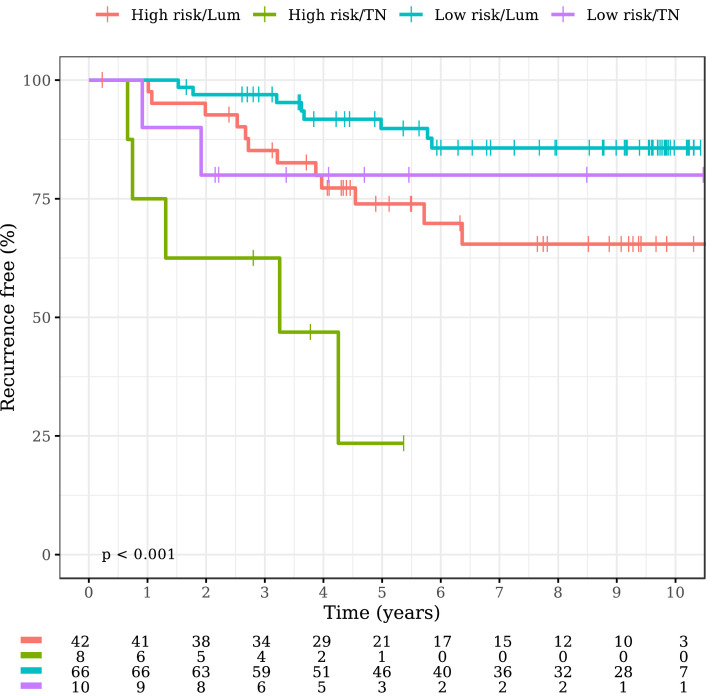
Kaplan-Meier plot of RFI by metabolomic score and Luminal and TN subtypes (HER2 positive subtype was omitted due to small numbers).

RFI was evaluated also by metabolomic score and molecular subtypes (luminal and TN). A significant difference was found between luminal tumors with high and low metabolomic risk (High risk/Lum vs. Low risk/Lum: HR 2.78, 95%CI 1.14–6.83, *p* = 0.025). The same trend was observed in patients with TN tumors (High risk/TN vs. Low risk/TN: HR 3.96, 95%CI 0.76–20.46, *p* = 0.101) (Fig. 4).

Finally, we exploratively evaluated RF risk score ability to predict BC-specific survival and OS. Unfortunately, in both cases, supervised analysis using the RF classifier on NMR data showed no separation (supplementary figure 4–5).

## Discussion

Clinicopathological prognostic factors commonly associated with increased risk of recurrence include large tumor size, nodal involvement, high tumor grade, negative hormone receptor status, HER2 overexpression, and the presence of lymph vascular invasion. These clinicopathological factors are combined within validated clinical tools (i.e. PREDICT tool [34], PORTRET tool [35]), to refine the prognostic risk estimation. Further refinement in risk stratification of eBC has been brought by gene-expression assays [11,12] which provide prognostic information by analyzing primary tumor tissues, and help in decisions regarding appropriate use of adjuvant chemotherapy in hormone receptor-positive/HER2-negative eBC. Despite the use of these prognostic tools, a significant proportion of patients classified as “high risk”, do not relapse and receive chemotherapy unnecessarily. Metabolomics has been proposed as a supportive prognostic tool that, deriving from the periphery (blood serum or plasma) instead of from the primary tumor tissue, has the potential to detect the presence of residual micrometastatic disease, and to capture the signal derived from the surrounding stroma and any host response to the tumor.

This retrospective analysis demonstrated that a metabolomic signature, identified employing NMR serum fingerprinting, had the ability to discriminate between early and advanced disease, in elderly patients with breast cancer. Metabolomics discriminated between early and metastatic groups with 81% sensitivity, 67% specificity and a predictive accuracy of 70% using NOESY spectra, implying that both low and high molecular weight molecules contribute to the discrimination between eBC and aBC. Metabolomics was also able to predict the risk of disease relapse in this patient population. The eBC patients who displayed a metabolomic profile resembling the metastatic disease, were classified as high risk, and had a higher probability to develop disease recurrence. Although age and menopausal status have emerged as crucial modulators of the global levels of circulating metabolites [36,37], and aging is often accompanied by mild or relevant comorbidities that have an impact on each individual metabolic profile [38], we obtained results comparable to the ones found in the other age-agnostic studies led by our group [16,18,19]. Very recently, our group has published the results of a study focused on elderly patients with early colorectal cancer. In this population, metabolomics was found to be a valuable tool to refine risk stratification and predict disease relapse [39]. The results observed in the present study, regarding the discrimination between eBC and aBC patients, are also in line with those of another research group [40]. They discriminated eBC and aBC patients using an OPLS model calculated on serum NMR spectra with 89.8% sensitivity, 79.3% specificity, and 84.1% accuracy, in a mostly postmenopausal population (mean age 56 and 57 years for eBC and aBC patients, respectively), with ER-positive HER2-negative breast cancer.

An examination of the individual metabolites that contributed to the discrimination of non-relapsed eBC as compared to aBC, was performed. Patients with metastatic disease were characterized by decreased levels of leucine, isoleucine, valine, acetate and histidine and increased levels of 3-hydroxybutyrate, citrate, N,N-Dimethylglycine, lipids and phenylalanine, as compared with patients with early disease who did not relapse. These results are in line with those produced by other studies [[18], [19], [40]], and are explainable considering that low histidine levels and high lipids are features linked with the metastatic progression, being associated with high tumor cell proliferation, high cell membrane turnover and lipid activity in intracellular signal transduction [41]. We also correlated the levels of individual metabolites with the risk of recurrence in the eBC cohort. However, none of them reached the statistical significance.

In our study, 66% of patients with eBC who experienced disease recurrence, were correctly classified as “high risk” from the metabolomic score. The metabolomic model showed a strong prognostic power, with “high risk” patients having a significantly increased probability of disease recurrence, as compared to patients with low risk metabolomic fingerprint (HR 3.42, 95% CI 1.58–7.37, *p*<0.001). We observed that the high risk metabolomic group, was slightly enriched in patients with known adverse clinicopathological prognostic factors, such as nodal involvement and tumor stage. Differently, tumor grading and molecular subtypes were equally distributed between high and low risk groups. In particular, TN tumors, that are commonly associated with a worse prognosis, were not enriched in the high risk metabolomic group.

The influence of these clinicopathological factors on the prognostic effect of the metabolomic signature, was evaluated within a multivariate analysis: the prognostic effect of the metabolomic classifier was independent of pathological N stage and pathological T stage. Also molecular subtypes did not interact with the metabolomic risk score, that was independently predictive of recurrence.

Similarly to genomic signatures, metabolomics can discriminate prognosis, independently from tumor stage, in older patients with luminal tumors. Of note, data from our group suggest that metabolomic prediction of risk recurrence could further split risk stratifications defined by Oncotype-DX alone [29]. Moreover, this study suggests that metabolomics is able to identify a subgroup of patients with TN tumors with a lower risk of disease recurrence, for whom it might be hypothesized that adjuvant chemotherapy could be de-escalated. Of course, the limited number of TN patients analyzed (*n* = 18), requires caution in the interpretation of the results.

Based on our data, we envisage two possible area of investigations of metabolomics in older BC patients. The benefit of adjuvant chemotherapy in older patients with high genomic risk ER+ eBC is still uncertain. It would therefore be interesting to evaluate the role of a metabolomic signature to further refine the risk of recurrence in this subgroup. This could lead to the identification of a subset of “high-high” risk patients in which evaluate the additional benefit of chemotherapy to endocrine therapy. Decision on the intensity of adjuvant chemotherapy in fit older patients with TN tumors is based on tumor stage with more intensive regimens used in high-risk patients. If confirmed in a larger series of older patients with TN eBC, our data could open the way to the evaluation of chemotherapy de-escalation in high clinical stage-low metabolomic risk patients.

To the best of our knowledge, this is the first study that has identified a metabolomic signature able to predict the risk of disease recurrence in an elderly BC cohort. The study has some limitations. Firstly, the limited number of patients with HER2-positive and TN eBC prevents firm considerations in these subgroups. Secondly, the relatively small number of BC events in the eBC cohort has prevented the possibility to observe a possible role of the metabolic signature in predicting BC-specific survival. Thirdly, medications taken by the patients were not considered in the present analysis and we are aware that exogenous factors affect metabolomic profiles. Finally, this was a monocentric study. The reproducibility of our data should be confirmed in a multicentric context and within a larger population in which all BC subtypes are well represented. The biochemical composition of biospecimens is affected by how samples are collected, stored, prepared and analyzed, and consequently differences in these steps can be particularly detrimental and affect the reproducibility of the model [42].The standardization of both pre-analytical and analytical procedures by universally adopting the specifications for metabolomics now available [43], has to be considered a mandatory step in future studies.

## Conclusions

Older patients with eBC require a personalized approach. Other causes of death can compete with breast cancer in determining patients’ outcome. Prognosticators that take into account not only the tumor, but also the host might be particularly relevant in the elderly. Our study suggests that a metabolomic signature might be of value in defining patients’ prognosis independently from classical clinicopathological features. Further studies are needed to confirm and validate the results obtained from our analysis, in order to enhance the role of NMR-based metabolomics in the management of older BC patients.

## CRediT authorship contribution statement

**Emanuela Risi:** Conceptualization, Data curation, Formal analysis, Investigation, Supervision, Validation, Visualization, Writing – original draft, Writing – review & editing. **Camilla Lisanti:** Conceptualization, Data curation, Project administration, Writing – original draft, Writing – review & editing. **Alessia Vignoli:** Data curation, Formal analysis, Methodology, Software, Supervision, Validation, Writing – original draft, Writing – review & editing. **Chiara Biagioni:** Data curation, Formal analysis, Investigation, Methodology, Software, Supervision, Validation, Writing – review & editing. **Agnese Paderi:** Investigation, Project administration, Supervision, Validation, Visualization, Writing – review & editing. **Silvia Cappadona:** Investigation, Methodology, Project administration, Supervision, Validation. **Francesca Del Monte:** Investigation, Methodology, Project administration, Supervision, Validation. **Erica Moretti:** Investigation, Project administration, Supervision, Validation, Visualization, Writing – review & editing. **Giuseppina Sanna:** Investigation, Project administration, Supervision, Validation, Visualization, Writing – review & editing. **Luca Livraghi:** Investigation, Project administration, Supervision, Validation, Visualization, Writing – review & editing. **Luca Malorni:** Conceptualization, Data curation, Formal analysis, Investigation, Supervision, Validation, Visualization, Writing – original draft, Writing – review & editing. **Matteo Benelli:** Data curation, Formal analysis, Investigation, Methodology, Software, Supervision, Validation, Writing – review & editing. **Fabio Puglisi:** Supervision, Validation, Visualization. **Claudio Luchinat:** Supervision, Validation, Visualization. **Leonardo Tenori:** Data curation, Formal analysis, Methodology, Resources, Software, Supervision, Validation, Writing – original draft, Writing – review & editing. **Laura Biganzoli:** Conceptualization, Data curation, Funding acquisition, Investigation, Project administration, Resources, Supervision, Validation, Visualization, Writing – original draft, Writing – review & editing.

## Declaration of Competing Interest

The authors declare that they have no known competing financial interests or personal relationships that could have appeared to influence the work reported in this paper.

## Appendix Supplementary materials

Image, application 1

Image, application 2

Image, application 3

Image, application 4

Image, application 5

Image, application 6
