# Supplementary material for: Risk assessment of disease recurrence in early breast cancer: A serum metabolomic study focused on elderly patients
Source: Transl Oncol. 2022 Nov 17;27:101585. doi: 10.1016/j.tranon.2022.101585 (PMC9676351; doi:10.1016/j.tranon.2022.101585)
Supplement: Supplementary file 1 [file mmc1.docx]

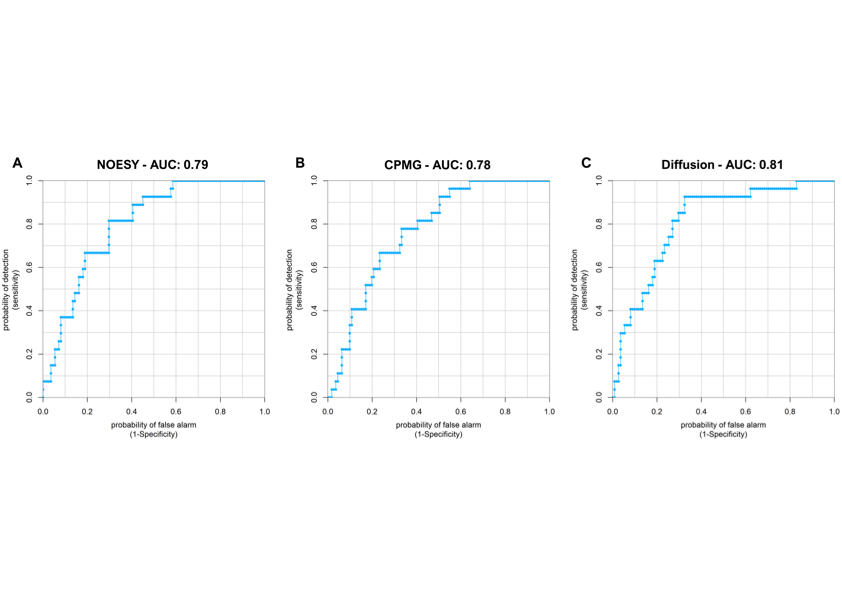


**Supplementary figure 1**: ROC curve analysis: discrimination between advanced breast cancer (aBC) and early breast cancer (eBC) free from disease recurrence (FFDR). the area under the curve (AUC) scores are presented for NOESY1D (A), CPMG (B) and DIFFUSION-edited (C).
