# Supplementary material for: Risk assessment of disease recurrence in early breast cancer: A serum metabolomic study focused on elderly patients
Source: Transl Oncol. 2022 Nov 17;27:101585. doi: 10.1016/j.tranon.2022.101585 (PMC9676351; doi:10.1016/j.tranon.2022.101585)
Supplement: Supplementary file 2 [file mmc2.docx]

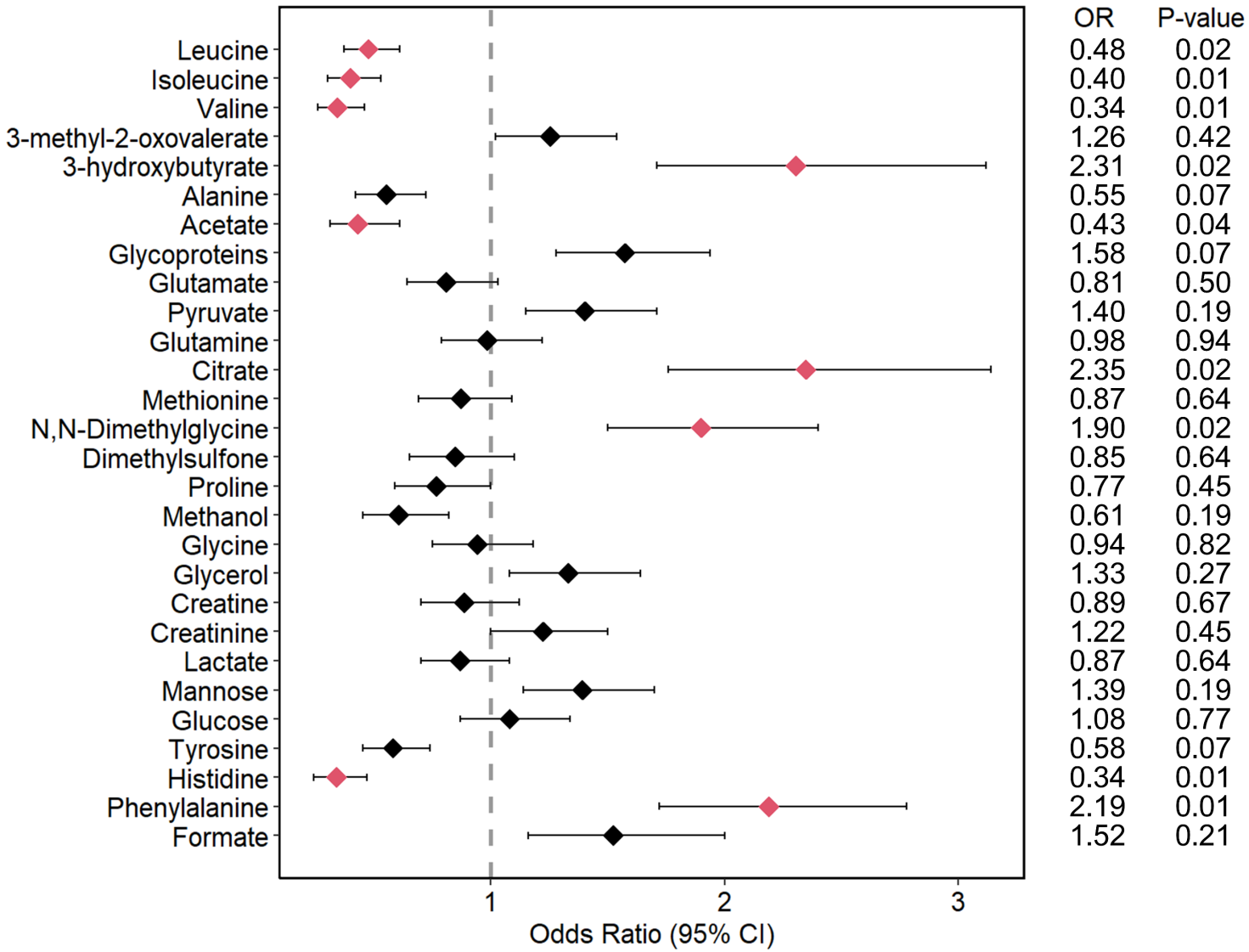


**Supplementary figure 2**: Forest plot of odds ratios (ORs) and 95% CI for metabolites. The center mark on each line corresponds to the OR estimate (red statistically significant, black not statistically significant) and the length of the line corresponds to the confidence interval (CI). ORs are adjusted for the time of serum sample acquisition. The early breast cancer (eBC) free from disease recurrence (FFDR) is the reference group, thus OR > 1 means higher level in advanced breast cancer (aBC), OR<1 means lower level in aBC. The p-values are adjusted for false discovery rate (FDR).
