# Supplementary material for: Risk assessment of disease recurrence in early breast cancer: A serum metabolomic study focused on elderly patients
Source: Transl Oncol. 2022 Nov 17;27:101585. doi: 10.1016/j.tranon.2022.101585 (PMC9676351; doi:10.1016/j.tranon.2022.101585)
Supplement: Supplementary file 3 [file mmc3.docx]

**Supplementary figure 3**: Forest plot of odds ratios (ORs) and 95% confidence interval (CI) for individual metabolites in the early breast cancer (eBC) cohort. The center mark on each line corresponds to the OR estimate, and the length of the line corresponds to the CI. ORs are adjusted for the time of serum sample acquisition. All metabolites have p-value > 0.05 in this comparison. The eBC free from disease recurrence (FFDR) is the reference group, thus OR >1 means higher level of that metabolite in relapsed-eBC patients, OR <1 means lower level of that metabolite in relapsed-eBC patients. Circled red marks refer to the metabolites statistically different in the comparison between eBC FFDR versus advanced breast cancer (aBC) patients.
