# Supplementary material for: Risk assessment of disease recurrence in early breast cancer: A serum metabolomic study focused on elderly patients
Source: Transl Oncol. 2022 Nov 17;27:101585. doi: 10.1016/j.tranon.2022.101585 (PMC9676351; doi:10.1016/j.tranon.2022.101585)
Supplement: Supplementary file 4 [file mmc4.docx]

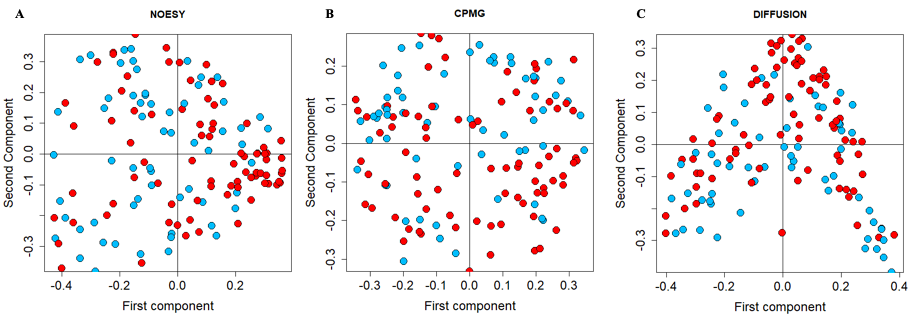


**Supplementary figure 4:** Breast cancer (BC)-specific survival prediction. Proximity plots of the random forest (RF) models discriminating survived patients (red, n=79), deceased patients (light blue, n=26) using: A) NOESY1D B) CPMG and C) DIFFUSION-edited nuclear magnetic resonance (NMR) spectra.
