# Supplementary material for: Risk assessment of disease recurrence in early breast cancer: A serum metabolomic study focused on elderly patients
Source: Transl Oncol. 2022 Nov 17;27:101585. doi: 10.1016/j.tranon.2022.101585 (PMC9676351; doi:10.1016/j.tranon.2022.101585)
Supplement: Supplementary file 6 [file mmc6.docx]

|  | **Low risk**  **N=84(%)** | **High risk**  **N=56(%)** | **p value** |
| --- | --- | --- | --- |
| **Age at study entry** |  |  | 0.101 |
| Median (Q1, Q3) | 75 (72, 79) | 77 (73, 81) |  |
| Range | 70 - 91 | 70 - 88 |  |
| **Pathologic T stage** |  |  | 0.295 |
| pT1 | 52 (62%) | 29 (52%) |  |
| pT2 | 32 (38%) | 27 (48%) |  |
| **Pathologic N stage** |  |  | 0.055 |
| N negative | 53 (63%) | 26 (46%) |  |
| N positive | 30 (36%) | 30 (54%) |  |
| Nx | 1 (1%) | 0 |  |
| **Vascular invasion** |  |  | 0.162 |
| Yes | 30 (36%) | 27 (48%) |  |
| No | 54 (64%) | 29 (52%) |  |
| **Histological types** |  |  | 0.227 |
| Ductal | 75 (89%) | 47 (84%) |  |
| Lobular | 4 (5%) | 7 (12%) |  |
| Other | 5 (6%) | 2 (4%) |  |
| **Histological grade** |  |  | 0.823 |
| G1 | 15 (18%) | 9 (16%) |  |
| G2/3 | 69 (82%) | 47 (84%) |  |
| **Estrogen receptor status (0% cutoff)** |  |  | 0.817 |
| ER negative | 13 (15%) | 10 (18%) |  |
| ER positive | 71 (85%) | 46 (82%) |  |
| **Progesteron receptor status (0% cutoff)** |  |  | 0.437 |
| PR negative | 20 (24%) | 17 (30%) |  |
| PR positive | 64 (76%) | 39 (70%) |  |
| **Her2 status (IHC)** |  |  | 1.000 |
| Her2 negative | 76 (90%) | 50 (89%) |  |
| Her2 positive | 8 (10%) | 6 (11%) |  |
| **Ki67 status (20% cutoff)** |  |  | 0.356 |
| Ki67<20 | 30 (36%) | 15 (27%) |  |
| Ki67>=20 | 54 (64%) | 41 (73%) |  |
| **Molecular type by IHC** |  |  | 0.871 |
| Luminal(ER+ and/or PR+ HER2-negative)** | 66 (79%) | 42 (75%) |  |
| Her2 positive | 8 (10%) | 6 (11%) |  |
| Triple negative | 10 (12%) | 8 (14%) |  |
| **Breast cancer event** |  |  | 0.003 |
| Yes | 10 (12%) | 19 (34%) |  |
| No | 74 (88%) | 37 (66%) |  |

T stage= tumor stage, N stage= node stage, IHC = immunohistochemistry, eBC= early breast cancer, ER= estrogen receptor, PR= progesterone receptor

**Supplementary table 1**: Descriptive table of eBC patient and tumor characteristics by metabolomic risk score.

*Other=no special type invasive carcinomas, mixed ductal-lobuar carcinomas

**ER+= ER ≥1%; PR+= PR ≥1%
